# Urban growth simulation and scenario projection for the arid regions using heuristic cellular automata

**DOI:** 10.1038/s41598-024-71709-4

**Published:** 2024-09-10

**Authors:** Xiaoyan Tang, Funan Liu, Xinling Hu

**Affiliations:** College of Civil Engineering and Architecture, Xinjiang University, Urumqi, 830046 China

**Keywords:** Gravitational search algorithm, Arid regions, Urban growth, Cellular automata, Scenario projection, Sustainability, Urban ecology

## Abstract

Arid regions tend to form compact urban patterns that have significant implications on urban growth and future urban patterns. Spatial simulation and projection using cellular automata (CA)-based models are important for achieving sustainable urban development in arid regions. In response to this need, we developed a new CA model (GSA-CA) using the gravitational search algorithm (GSA) to capture and project urban growth patterns in arid regions. We calibrated the GSA-CA model for the arid city of Urumqi in Northwest China from 2000 to 2010, and validated the model from 2010 to 2020, and then applied to project urban growth in 2040. The results indicated that the optimal performance of the model was achieved when the fraction of the population was 0.5. GSA-CA achieved an overall accuracy of 98.42% and a figure of merit (FOM) of 43.03% for the year 2010, and an overall accuracy of 98.52% with FOM of 37.64% for 2020. The results of the study help to adjust urban planning and development policies. The developed model has the potential to be employed in simulating urban growth and future scenarios in arid regions globally, including Northwest China and Africa.

## Introduction

Understanding urban growth in arid regions is critical to sustainable development, especially in regions such as Northwest China and Central Asia^1^. These areas face special geographical and climatic environmental challenges, leading to scarcity of built-up land, scarce precipitation, sparse vegetation, and vulnerability to natural disasters including droughts, dust storms, and sandstorms^2^. Topography, climate, and environmental factors constrain urban growth in arid regions, and thus urban patterns in these regions typically form compact patterns^3,4^. Therefore, it is critical to accurately simulate and project urban growth using multiple spatial datasets and simulation models. Dynamic modeling can help mitigate the adverse environmental, economic, and social impacts of urban growth and thus provide important information for urban planning^5,6^. Cellular automata (CA) models are an effective tool for capturing the spatial and temporal variability of complex geographic phenomena, including urbanization^7,8^. Although CA models have been widely used globally to simulate future urban growth scenarios, the major applications have been focused on densely populated and relatively flat coastal areas and relatively little attention has been paid to less populated arid regions^9^. To address this gap, it is a challenge for current CA research to construct more appropriate simulation models for urban growth in arid regions, taking into account topographical factors and arid climates to adjust urban development strategies for sustainable development.

The simulation of urban growth using CA models depends on transformation rules, which directly affect the results of their simulation. The establishment of rules is based on a variety of methods, including statistical regression, fuzzy logic, decision trees, and various forms of artificial intelligence^10–12^. Heuristic algorithms are a branch of artificial intelligence methods that are increasingly used in the CA modeling of urban growth. Typical heuristic algorithms include generic heuristics, evolutionary heuristics, and swarm heuristics^13,14^. Of these, typical swarm heuristic algorithms include particle swarm optimization, ant colony optimization, and gravitational search algorithm, which have demonstrated superior accuracy and spatial distribution in the simulation of land use and urban growth^15–17^. However, given the diversity of heuristic algorithms and differences in control parameters, CA models based on different heuristic algorithms yield different simulation results and apply to different regions. Therefore, for arid regions such as Urumqi in Northwest China, it is imperative to enhance the simulation and projection of urban growth in these regions by incorporating climatic variables such as drought to guarantee the results are accurate and reflect the distinctive environment and development dynamics.

Researchers have developed and validated different CA models for simulating and projecting urban growth^18–20^. However, these models were usually designed for fast-growing coastal areas and are not universally applicable to slower-growing arid regions^21^. Therefore, these models need to be modified when applied to the arid regions of Northwest China. The arid regions of Northwest China have complex topography and comparatively slow growth rates. Specifically, there are two main characteristics: (1) the undulating terrain restricts urban growth, and the land-use type is mostly desert, which makes the construction land scarce, and thus it is necessary to consider the terrain factor in the CA model; and (2) the low rainfall and high evapotranspiration rate lead to sparse vegetation, which are important considerations for the urban growth model. This suggests that developing CA models appropriate for arid regions is a pressing necessity to explore the spatio-temporal patterns for urban growth in Northwest China. These models will greatly improve the planning optimization and carrying capacity of urban layouts in these regions^22,23^.

In light of the aforementioned considerations, CA model development and projection research for arid areas in Northwest China, such as Yinchuan City, Xining City, and Urumqi City, urgently needs to be strengthened. This study aims to address several key research questions for urban simulation in arid regions: (1) Can heuristics effectively simulate urban growth in typical arid regions? (2) Can the parameters of the heuristic algorithm be adjusted to more accurately simulate urban growth in arid regions? (3) Can the resulting models accurately project future urban growth scenarios for arid cities in Northwest China, such as Urumqi? The insights gained from answering these questions will greatly enhance the CA modeling framework and contribute to a deeper understanding of urban growth in arid regions, especially in Northwest China. The gravitational search algorithm (GSA) utilizes Newton’s Law of Gravity and the Second Law of Motion to avoid local optimization and enhance the global search capability by dynamically adjusting the gravitational force and acceleration to converge rapidly; simultaneously, due to the effect of gravitational force, objects with large mass are given priority, which can make the solution set converge towards better solutions, thus improving the overall search accuracy; moreover, compared with other complex optimization algorithms, the algorithm structure is simple which requires excessive parameter adjustments and is easy to implement^17^. Consequently, we constructed a CA model (named GSA-CA) using GSA, and the model was developed within the UrbanCA framework^24^. This framework incorporates a time-increasing parameter and a locally adjusted parameter for generating the probability of occurrence map. The GSA-CA model was used to investigate the dynamics of urban growth and to project future urban scenarios for the urban area of Urumqi, situated in Northwest China. In our study, we calibrated the GSA-CA model with data from 2000 to 2010, validated the model for 2010–2020, and projected the urban scenarios for 2040 for Urumqi. Through this study, we endeavor to develop a new approach for urban growth in arid regions and apply it to different arid regions.

## Methods

### The workflow

**Fig. 1:**
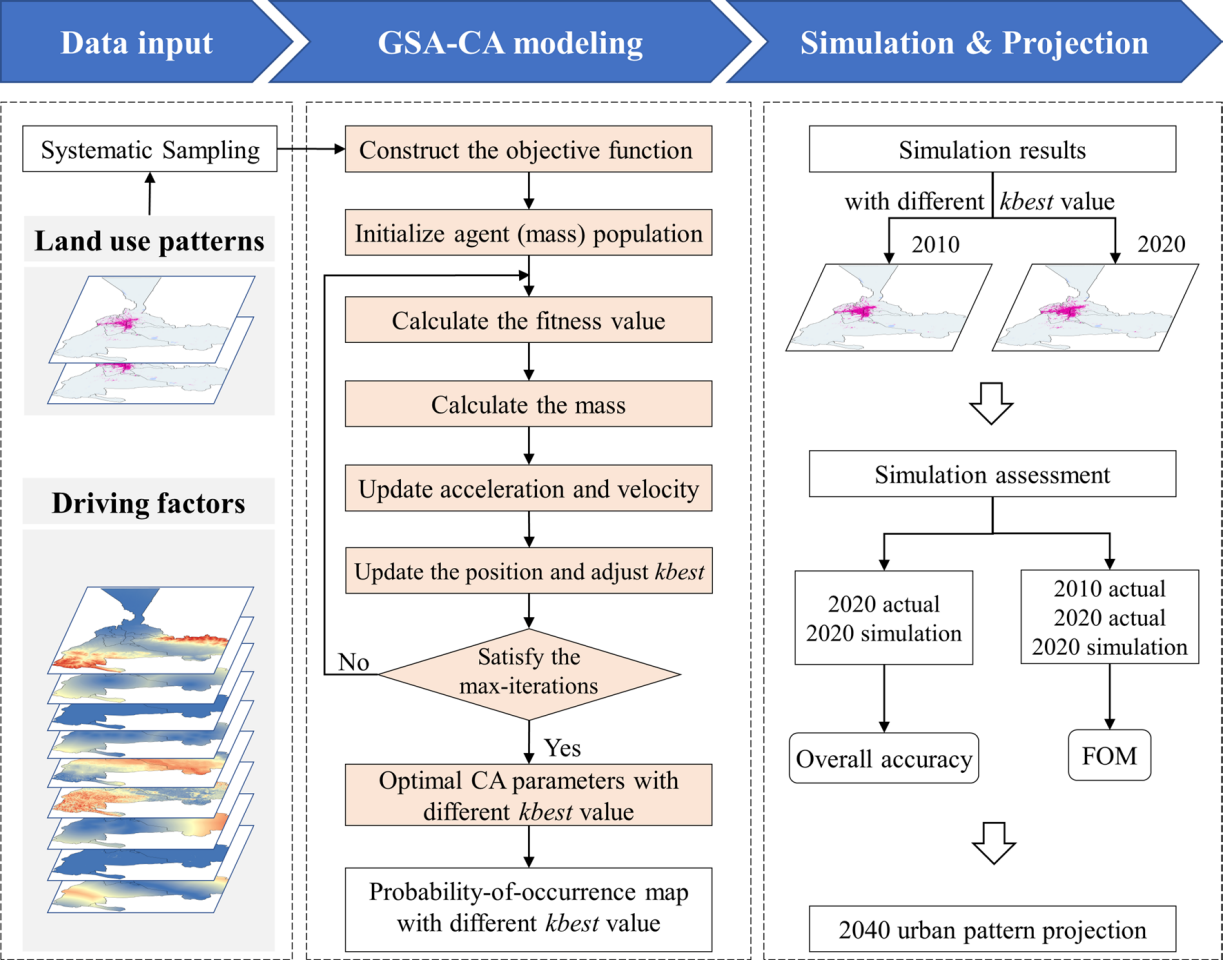
Workflow of the GSA-CA model for the simulation and projection of dynamic urban growth.

Figure 1 shows how to optimize CA parameters using the heuristic GSA for constructing a dynamic simulation model to project urban growth in an arid area. We selected nine driving factors for modeling, ranging from topography, human impact, socio-economics, and climate. We used a systematic sampling method to extract training samples from the input land-use maps (2000 and 2010) and the factor layer (i.e., the 9 factors). We then constructed an objective function representing the difference between the GSA-CA model and the actual urban growth, projecting the space of urban growth models into the heuristic GSA's search space. Based on the objective function, we initialized the agent population size, calculated the fitness function value and mass, and updated the optimal position. Finally, GSA identified the optimal CA parameters and generated the probability of occurrence maps using GSA-CA with different fractions of the population with the best fitness. Ultimately, the GSA-CA model was used to project two urban growth scenarios for Urumqi in 2040. Modeling and implementation were performed in UrbanCA software, which is available to users worldwide^24^.

### The basic urban CA model

CA can be considered as a state model consisting of a cell space and a transformation function that defines the likelihood of urban growth. In the CA framework, the transformation rules are established through the combination of five factors: cell state (*CST*), urban growth factor (*P*_*UGR*_), neighborhood effect (*NEF*), spatial constraints (*SCO*), and stochastic factors (*SFA*)^25^. The CA transformation rules can be expressed as^26^:

1 $$CST_{i}^{t + 1} = TFU\left( {CST_{i}^{t} ,P_{UGR} ,NEF,SCO,SFA} \right)$$

where $CST_{i}^{t + 1}$ and $CST_{i}^{t}$ denote the state of cell *i* at time *t* and *t* + *1*, respectively; *TFU* denotes the transformation function.

The global probability of occurrence (*P*_*GPO*_) can be given by^24,27^:

2 $$\left\{ {\begin{array}{*{20}c} {P_{GPO} = \frac{{(P_{UGR} \times \left( {1 + S_{TIP} )^{t - 1} + P_{NEF} \times S_{LAP} } \right) \times SCO \times SFA}}{2}} \\ {P_{UGR} = \frac{{e^{{a_{0} + a_{1} \times x_{1} + a_{2} \times x_{2} + \cdots + a_{j} \times x_{j} + \delta }} }}{{1 + e^{{a_{0} + a_{1} \times x_{1} + a_{2} \times x_{2} + \cdots + a_{j} \times x_{j} + \delta }} }}} \\ \end{array} } \right.$$

where *S*_*TIP*_ denotes a time-increment parameter to resist the decaying effect of local probability-of-occurrence; *S*_*LAP*_ denotes a local adjustment parameter to reduce the enhancement of the neighborhood effects; *a*_*0*_ denotes a constant; x_*j*_ denotes the *j-*th driving factor of urban growth; a_*j*_ denotes the weight of factor x_*j*_; *δ* denotes the modeling residual.

Smaller modelling residuals can lead to optimal CA parameters, which improve the simulation accuracy. To obtain these parameters with reduced modelling residuals, the objective function can be given by^24^:

3 $$F\left( {\varvec{u}} \right) = \sqrt {\frac{{\mathop \sum \nolimits_{i}^{s} \left( {P_{GPO} \left( {\varvec{u}} \right) - P_{0} } \right)_{i}^{2} }}{s} }$$

where *F(u)* denotes the objective function; *u* = (*u*_*0*_*, …*, *u*_*d*_) denotes a feasible solution of CA parameters; *P*_*0*_ denotes the observed urban growth; *s* denotes the number of samples.

### The GSA-CA model

To realize the objective function, we employ the GSA method originally proposed by^17^, which searches for the optimal solution through iterations. It is assumed that there are *n* agents (masses) in a *d*-dimensional search space, and each agent represents a unique set of feasible CA parameters, with each dimension associated with a factor that affects urban growth. The configuration of agents (masses) can be characterized as:

4 $$Z_{k} = \left( {F\left( {\varvec{u}} \right),\left( {u_{0} , \ldots ,u_{d} } \right)_{k} ,A_{k}^{t + 1} } \right)$$

where $A_{k}^{t + 1}$ denotes the position of the *k-*th agent selected (the *k-*th CA parameter) at the* t* + *1*-th iteration.

A higher mass leads to a more efficient agent that walks slower and has a higher attraction. We update the mass ($M_{k}^{t}$) by the following formula:

5 $$\left\{ {\begin{array}{*{20}c} {M_{k}^{t} = \frac{{m_{k}^{t} }}{{\mathop \sum \nolimits_{k = 1}^{n} m_{k}^{t} }}} \\ {m_{k}^{t} = \frac{{F\left( u \right)_{k}^{t} - Max_{{k \in \left\{ {1, \cdots ,n} \right\}}} F\left( u \right)_{k}^{t} }}{{Min_{{k \in \left\{ {1, \cdots ,n} \right\}}} F\left( u \right)_{k}^{t} - Max_{{k \in \left\{ {1, \cdots ,n} \right\}}} F\left( u \right)_{k}^{t} }}} \\ \end{array} } \right.$$

where $m_{k}^{t}$ represents the inertial mass of agent *k* at time *t*; $F\left( u \right)_{k}^{t}$ represents the objective function value of the *k-th* agent at time *t*; $Max_{{j \in \left\{ {1, \ldots ,N} \right\}}} F\left( u \right)_{k}^{t}$ and $Min_{{j \in \left\{ {1, \cdots ,N} \right\}}} F\left( u \right)_{k}^{t}$ correspond to the maximum and minimum values of the objective function value, respectively; *n* represents the number of agents.

According to the law of motion, the acceleration *a* of agent *k* at time *t* ($a_{k}^{t}$) is calculated as follows:

6 $$\left\{ {\begin{array}{*{20}c} {a_{k}^{t} = \frac{{F_{k}^{t} }}{{M_{k}^{t} }}} \\ {F_{k}^{t} = \mathop \sum \limits_{l = 1,l \ne k}^{N} rand_{l} F_{kl}^{t} } \\ {F_{kl}^{t} = G\left( t \right)\frac{{M_{pl}^{t} \times M_{al}^{t} }}{{R_{kl}^{t} + \varepsilon }}\left( {A_{l}^{t} - A_{k}^{t} } \right)} \\ {R_{kl}^{t} = ||A_{k}^{t} ,A_{l}^{t}||_2 } \\ \end{array} } \right.$$

where $F_{k}^{t}$ and $F_{kl}^{t}$ represents the total force acting on agent k and the force acting on l-th mass from *k-th* mass, respectively; *rand*_*l*_ is a random number in the interval [0,1]; *G(t)* is gravitational constant at time *t*; $M_{al}^{t}$ and $M_{pl}^{t}$ is the active gravitational mass and passive gravitational mass related to agent l, respectively; e is a small constant; $R_{kl}^{t}$ is the Euclidian distance between two agents *k* and *l*; $A_{k}^{t}$ and $A_{l}^{t}$ denote position of *k-th* and *l-th* agent at time *t*, respectively.

The velocity of the agent is considered to be the sum of its current velocity and acceleration; thus the position of the agent is updated with the following equation:

7 $$\left\{ {\begin{array}{*{20}c} {v_{k}^{t + 1} = rand_{k} \times v_{k}^{t} + a_{k}^{t} } \\ {A_{k}^{t + 1} = A_{k}^{t} + v_{k}^{t + 1} } \\ \end{array} } \right.$$

where $v_{k}^{t + 1}$ and $v_{k}^{t}$ denote velocity of *k-th* agent at time *t* and *t* + *1*, respectively; $A_{k}^{t + 1}$ denote the position of *k-*th agent at time *t* + *1*.

### The model evaluation methods

To evaluate the accuracy of the simulation results, an error matrix was computed to enable a cell-by-cell comparison of the observed urban patterns with those simulated by the GSA-CA model^28^. The matrix yields two key metrics: overall accuracy (OA) and figure of merit (FOM).

## Study area and data sources

### Study area

**Fig. 2:**
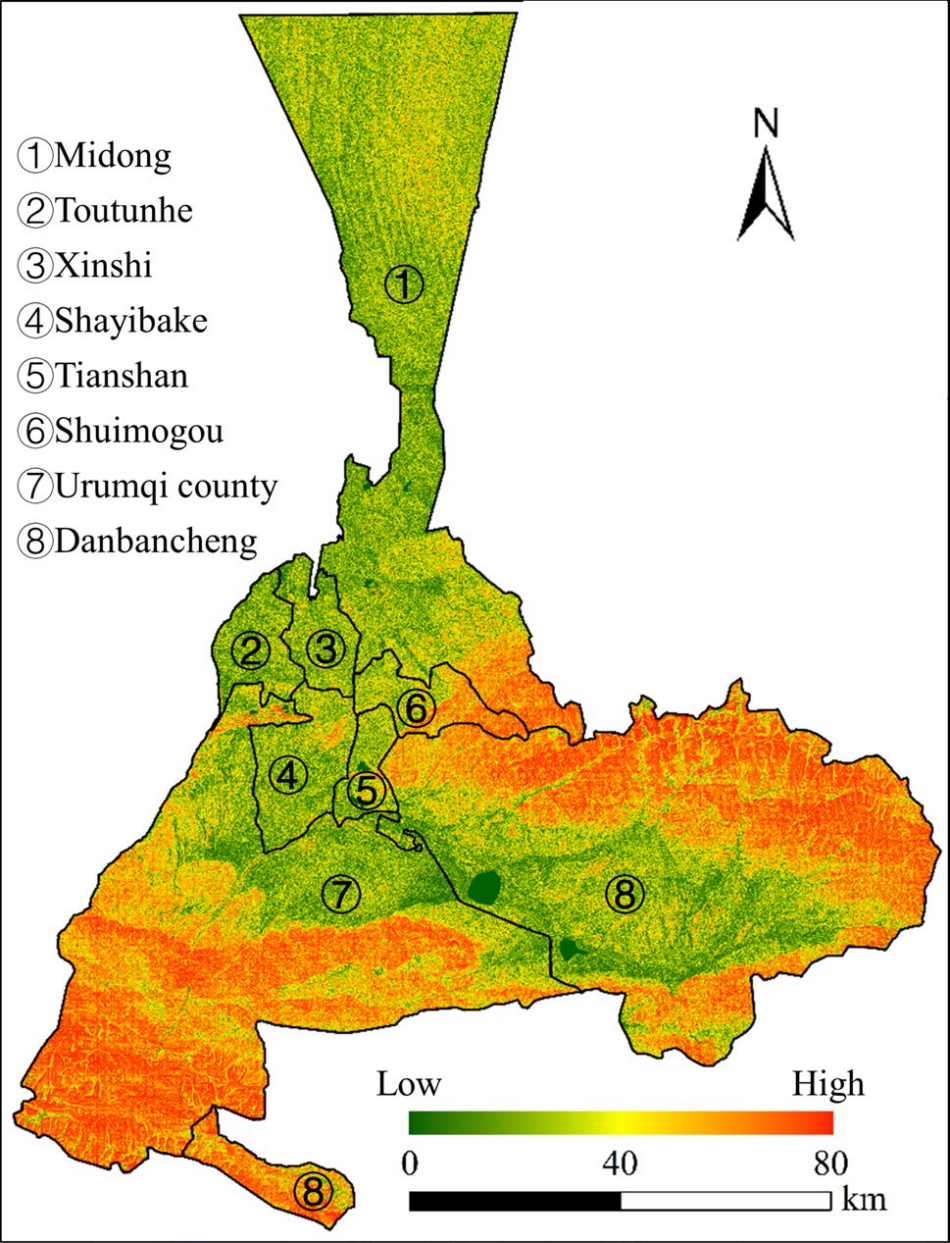
The administrative profile of the study area with slope. The map is created by ArcMap, version 10.8 (http://www.esri.com).

Urumqi is a typical arid city in Northwest China, and the study of its urban growth can provide a reference for urban layout planning in such areas. The city is the capital of Xinjiang Uygur Autonomous Region in Northwest China and has a continental temperate climate. The average annual precipitation of the city is 236 mm, and evapotranspiration is much greater than precipitation, resulting in sparse vegetation, making the drought as a major constraint for urban growth. Urumqi had a population of 4.08 million in 2023, of which more than 90 percent was made up of urban dwellers. The city is encircled by mountains on three sides and has seven districts and one county with a total area of 13,800 square kilometers (urumqi.gov.cn). The Xinshi District, Tianshan District, and Shayibake District, along with the surrounding areas are the center of urban growth in Urumqi (Fig. 2).

### Datasets and the preprocessing

We used GLC_FCS30 datasets with a resolution of 30 m as land use data for the years 2000, 2010, and 2020^29^. The official overall accuracy of GLC_FCS30 as a public land use data product is 82.5%, with a kappa coefficient of 0.784^29^. Additionally, this data product performs exceptionally well in capturing intricate urban details in arid regions^30^. We combined the GLC_FCS30 datasets into three types: urban, nonurban, and excluded types (water body), as our study focuses on urban growth in arid regions. Studies have shown that urban growth is influenced by topography, human activities, climate change, and socio-economics^31,32^. Among these factors, we selected nine influence factors to characterize the arid regions and express their relationship with urban growth. We used the Shuttle Radar Topography Mission Digital Elevation Model (SRTM DEM) to calculate surface elevations to assess the impact of topography on urban growth. We extracted the spatial proximity of city centers, town centers, railways, and roads, and assessed their impact as human influences features on urban growth. Given the study area is located in arid regions, we chose drought intensity and land surface temperature (LST) as climatic factors to assess their impact on urban growth. Among them, drought intensity^33^ was calculated based on the LNPS-EWM model using the entropy weighting method and considering four factors including land surface temperature, normalized difference vegetation index, potential evapotranspiration, and soil moisture. Specifically, we pre-processed the above-mentioned four factors, including land surface temperature downscaling, obtaining soil moisture and validation. The factor weights determined using the entropy weighting method can objectively reflect the information of the original data, thus effectively avoiding the bias caused by human factors. Consequently, these factors were normalized to be dimensionless, and the information entropy for each factor was calculated based on the entropy weight method to determine the weight of each factor in the LNPS-EWM model to generate the drought intensity map. Socioeconomic factors include gross domestic product (GDP) from the NOAA website and population per pixel (PPP) from the worldpop website (Table 1).

**Table 1:** Influence factors used to examine the spatiotemporal pattern of urban growth.

Type	Category	Dataset	Scale (m)	Source
Raster	Topography	DEM	30	earthexplorer.usgs.gov
Point	Human influences	City	–	openstreetmap.org
Point	Human influences	Town	–	openstreetmap.org
Line	Human influences	Railway	–	openstreetmap.org
Line	Human influences	Road	–	openstreetmap.org
Raster	Climate	Drought intensity	500	The entropy weight method
Raster	Climate, land surface	LST	1000	geodata.cn
Raster	Socioeconomic	GDP	1000	ngdc.noaa.gov
Raster	Socioeconomic	PPP	100	worldpop.org

**Fig. 3:**
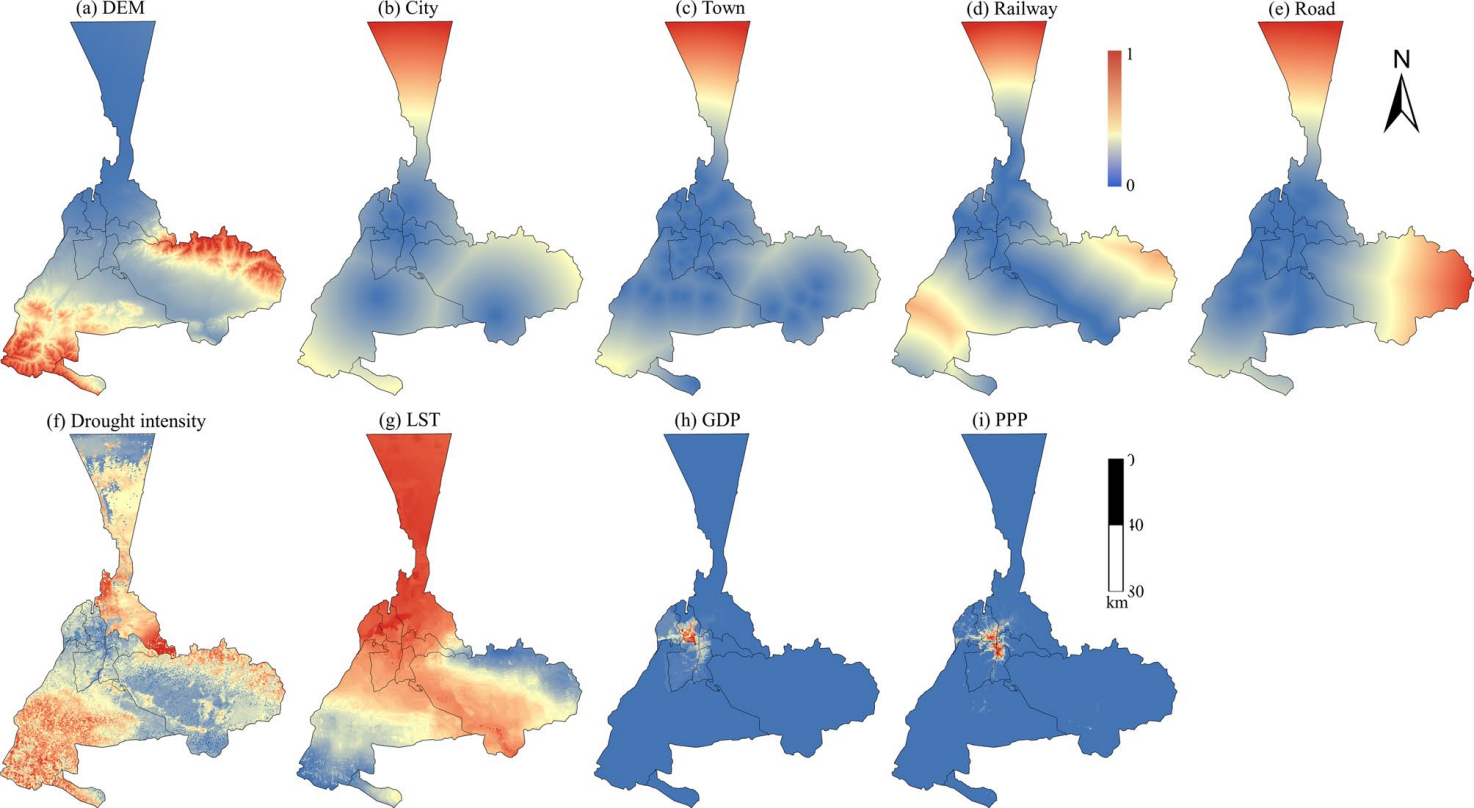
Normalized spatial variables for modeling dynamic urban growth in Urumqi. The map is created by ArcMap, version 10.8 (http://www.esri.com).

To reduce the computational cost and speed up the simulation process, we normalized all the spatial variables. The normalized proximity variables and remotely sensed images were then visualized as input layers for the CA model using ArcGIS 10.8 (Fig. 3).

## Results

### Observed urban growth pattern

**Fig. 4:**
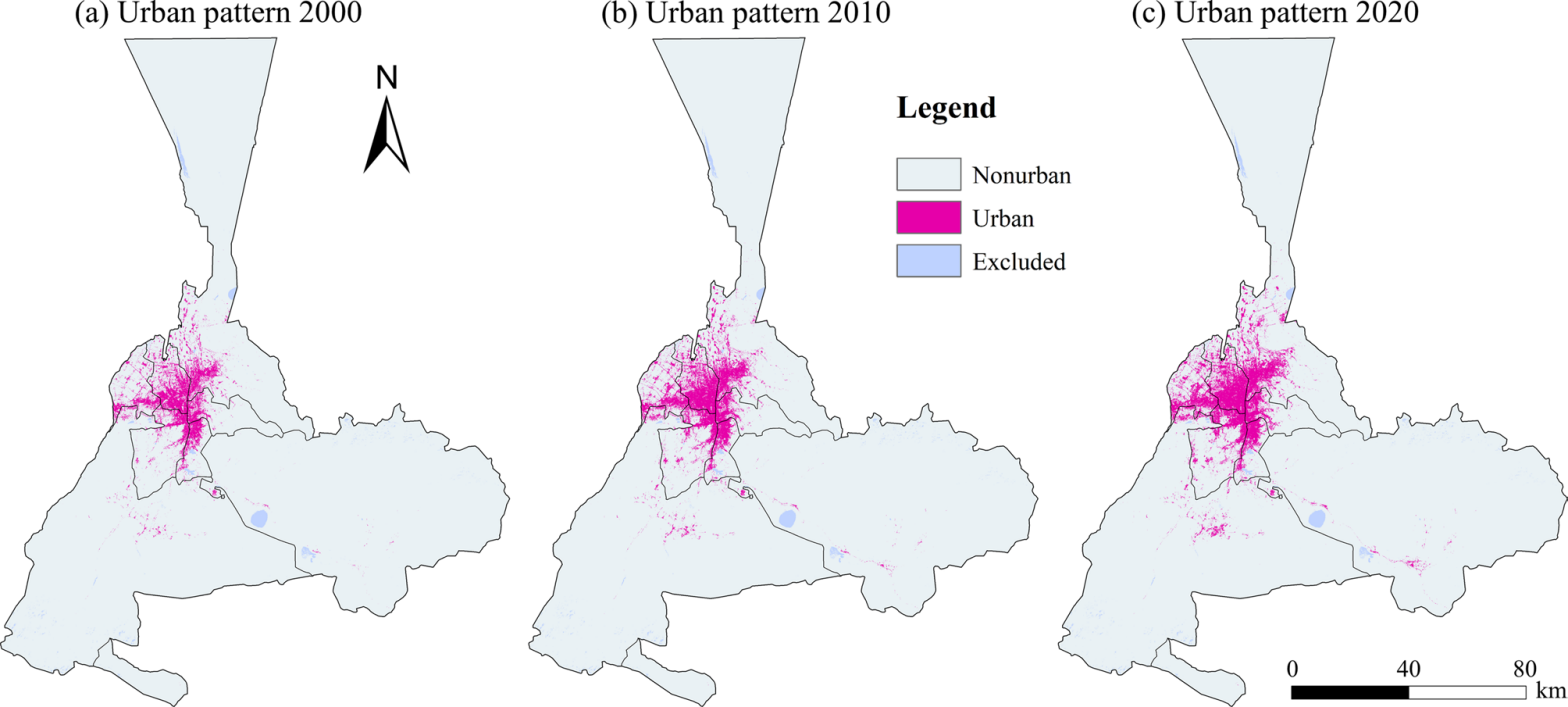
Urban patterns in 2000, 2010, and 2020 for Urumqi. The map is created by ArcMap, version 10.8 (http://www.esri.com).

To analyze urban growth in Urumqi, we collected multiple types of GLC_FCS30 for the years 2000, 2010, and 2020 to generate urban patterns for Urumqi (Fig. 4). Here, we considered impervious surfaces as urban and water body as a spatial constraint on urban growth, which was excluded from further modeling, and then merged the remaining land use categories as non-urban. Between 2000 and 2010, urban growth in Urumqi occurred mainly in low-lying areas close to the existing built-up areas, showing wrap-around growth (Figs. 4a, b). Between 2010 and 2020, urban growth in Urumqi occurred mainly in the northeast and southwest of the original built-up area, in the Xinshi District, Tianshan District, Shaybak District, and the surrounding low-lying areas, showing a significant agglomeration pattern (Fig. 4c).

### The CA parameters and probability of occurrence map

The optimal solution of a heuristic algorithm is vulnerable to its control parameters. Among heuristic algorithms like GSA, smaller populations (*numPopulation*) may lead to locally optimal solutions, while larger populations may lead to heavy computational loads. In this study, *numPopulation* was specified as 20 times the number of variables according to an earlier publication^24^. The change in the fraction of the population (*kbest*) leads to different simulation results. In the study, we determined the optimal *kbest* through running simulations with *kbest* set at 0.1, 0.2, 0.3, 0.4, 0.5, 0.6, 0.7, 0.8, and 0.9, respectively. We set the lower bounds for all positive parameters and the upper bounds for all negative parameters to zero. Instead, the upper bounds of the positive parameters and the lower bounds of the negative parameters were set to twice the parameters obtained from logistic regression^24^. We fixed the number of iterations (*maxIter*) to 5000 and the tolerance threshold to 1e−6 (Table 2).

**Table 2:** The GSA control parameters for retrieving the CA transformation rules.

Controlling setting	Parameter	Implication
*optimType*	MIN	Minimization of the objective function
*rangeVar*	$$\left[ {\begin{array}{*{20}c} { - 20,} \\ {0,} \\ \end{array} \begin{array}{*{20}c} { - 27,} \\ {0,} \\ \end{array} \begin{array}{*{20}c} {0,} \\ {20.5,} \\ \end{array} \begin{array}{*{20}c} {0,} \\ {2,} \\ \end{array} \begin{array}{*{20}c} { - 0.5,} \\ {0,} \\ \end{array} \begin{array}{*{20}c} { - 2.5,} \\ {0,} \\ \end{array} \begin{array}{*{20}c} { - 20,} \\ {0,} \\ \end{array} \begin{array}{*{20}c} { - 20,} \\ {0,} \\ \end{array} \begin{array}{*{20}c} {0,} \\ {18,} \\ \end{array} \begin{array}{*{20}c} { - 60} \\ 0 \\ \end{array} } \right]$$	The lower bound (minimum) and upper bound (maximum) values of the ten variables, respectively
*numPopulation*	200	Population sizes to determine the number of populations
*maxIter*	5000	The maximum number of iterations
*kbest*	0.1, 0.2, 0.3, 0.4, 0.5, 0.6, 0.7, 0.8, 0.9	positive numeric between 0 and 1 to determine the fraction of the population with the best fitness
*Convergence tolerance*	1e−6	Differences in acceptable function value

We used the GSA to obtain CA parameters for different fractions of the population with the best fitness scenarios (Table 3). Except for GDP and PPP, positive values of the parameters for the other factors indicate an inhibitory effect on urban growth, while negative values indicate a facilitating effect on urban growth. Moreover, the absolute value of each parameter reflects the extent of its impact on urban growth. In the GSA-CA model, DEM and LST are the most important factors inhibiting urban growth, and town and city are the most significant factors contributing to urban growth.

**Table 3:** Generated CA parameters using GSA-CA for different fractions of the population with the best fitness scenarios.

Variable	GSA-CA with different fractions of the population *(kbest)*								
	0.1	0.2	0.3	0.4	0.5	0.6	0.7	0.8	0.9
Constant	− 8.89	− 8.89	− 8.70	− 8.44	− 8.35	− 8.36	− 9.21	− 8.27	− 8.74
DEM	15.76	15.75	15.52	15.19	14.96	14.56	14.94	12.70	12.27
City	− 18.41	− 18.42	− 18.72	− 19.72	− 19.43	− 18.78	− 16.97	− 16.01	− 15.55
Town	− 58.30	− 58.17	− 55.39	− 48.05	− 41.38	− 37.35	− 30.78	− 33.45	− 33.51
Railway	− 12.57	− 12.56	− 12.54	− 12.93	− 12.89	− 12.22	− 11.07	− 10.62	− 11.08
Road	− 15.65	− 15.70	− 16.03	− 13.79	− 13.85	− 13.34	− 11.67	− 10.19	− 11.04
Drought intensity	1.62	1.62	1.60	1.50	1.38	1.26	1.06	0.96	0.88
LST	7.91	7.90	7.69	7.34	7.18	7.17	7.89	7.22	8.18
GDP	− 0.24	− 0.24	− 0.25	− 0.25	− 0.22	− 0.17	− 0.01	− 0.05	− 0.29
PPP	− 1.77	− 1.77	− 1.73	− 1.58	− 1.47	− 1.39	− 1.20	− 1.25	− 1.32

**Fig. 5:**
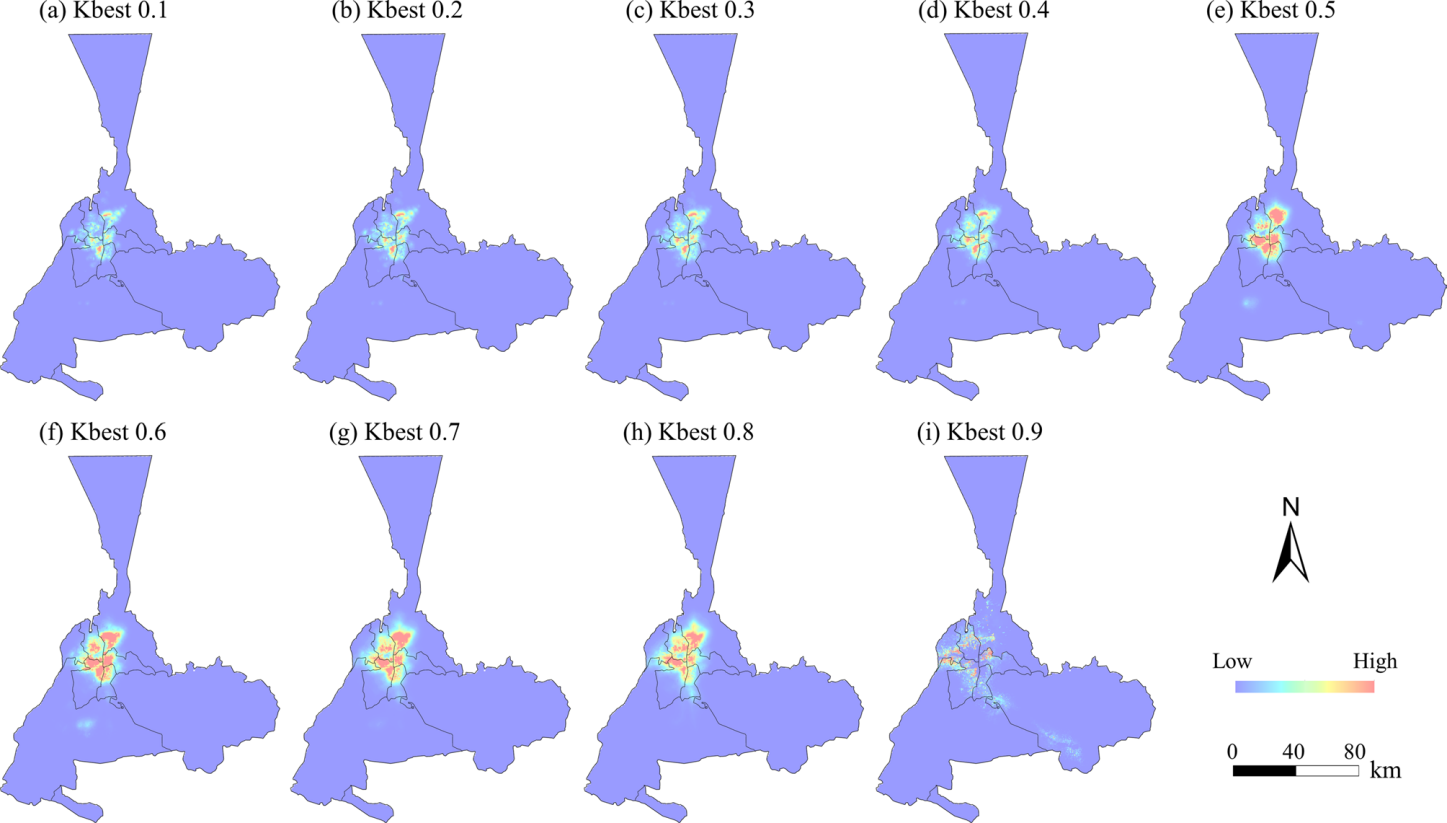
The probability of occurrence maps retrieved by CA parameters. The map is created by ArcMap, version 10.8 (http://www.esri.com).

Figure 5 shows the probability of occurrence maps generated using the GSA-CA model for different *kbest*, with the probability of occurrence values ranging from 0 to 1. Visual inspection of the probability of occurrence maps shows higher probabilities around the city center, suggesting that land close to the existing city center is more suitable for development. Significantly, the probability of occurrence map for *kbest* settings of 0.5 was slightly higher than those for other *kbest* values, suggesting that *kbest* of 0.5 was likely to be the most efficient simulation scale. The probability of occurrence maps were used to construct a CA model to simulate the urban pattern in 2020 and project future urban growth in 2040.

### The simulated results

**Fig. 6:**
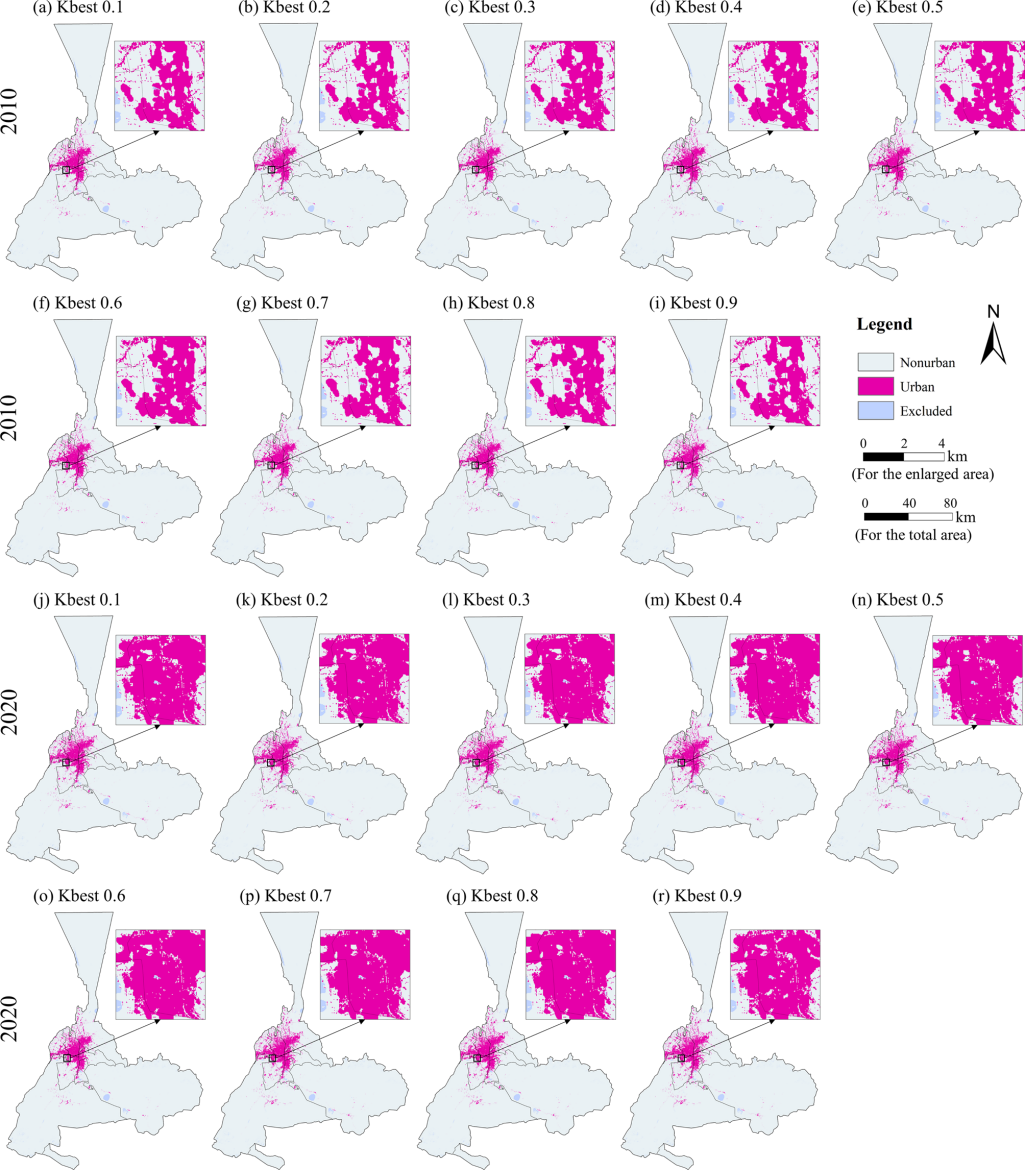
The simulated urban patterns using the GSA-CA model with different fractions of the population for 2010 and 2020. The map is created by ArcMap, version 10.8 (http://www.esri.com).

We used the GSA-CA model to simulate the urban pattern for 2010 (Figs. 6a–i) and 2020 (Figs. 6j–r). The results showed that the urban growth from 2000 to 2020 mainly concentrated in the low-lying areas in proximity to the existing built-up areas, demonstrating a tendency towards outward urban growth. Urumqi's urban growth primarily occurs in the Xinshi District, Tianshan District, Shaybak District, and the surrounding low-lying areas, exhibiting a clear agglomeration pattern.

### The GSA-CA model assessment

**Fig. 7:**
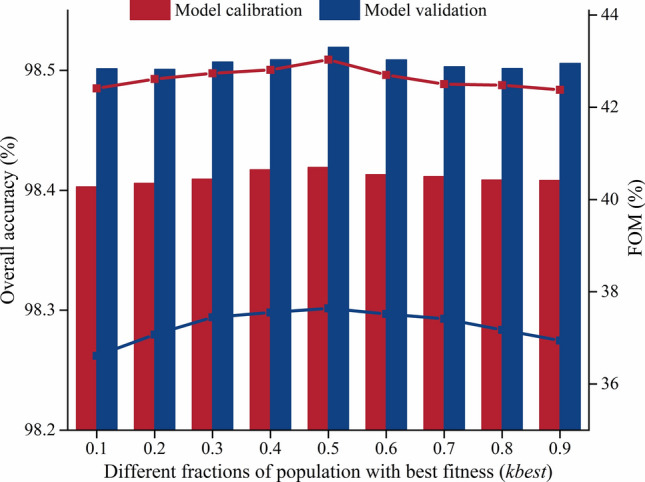
Overall accuracy and FOM during the model calibration and validation under different fractions of the population (*kbest*) for 2010 and 2020.

A pixel-by-pixel comparison was conducted between the simulated urban growth and the actual growth observed for the years 2010–2020. Figure 7 illustrates that the simulation accuracy increased and then decreased as the fractions of the population (*kbest)* value increased. The overall accuracy of the urban pattern simulations in both the calibration and validation phases exceeded 98.40%, with the FOM exceeding 36.50%. During the calibration phase, the highest overall accuracy (98.42%) and FOM (43.03%) were observed at *kbes*t value of 0.5. A similar pattern was observed in the validation phase, with peaks in overall accuracy (98.52%) and FOM (37.64%). These results indicated that the GSA-CA model performed well and had a slightly higher simulation capability in the calibration phase than in the validation phase.

**Fig. 8:**
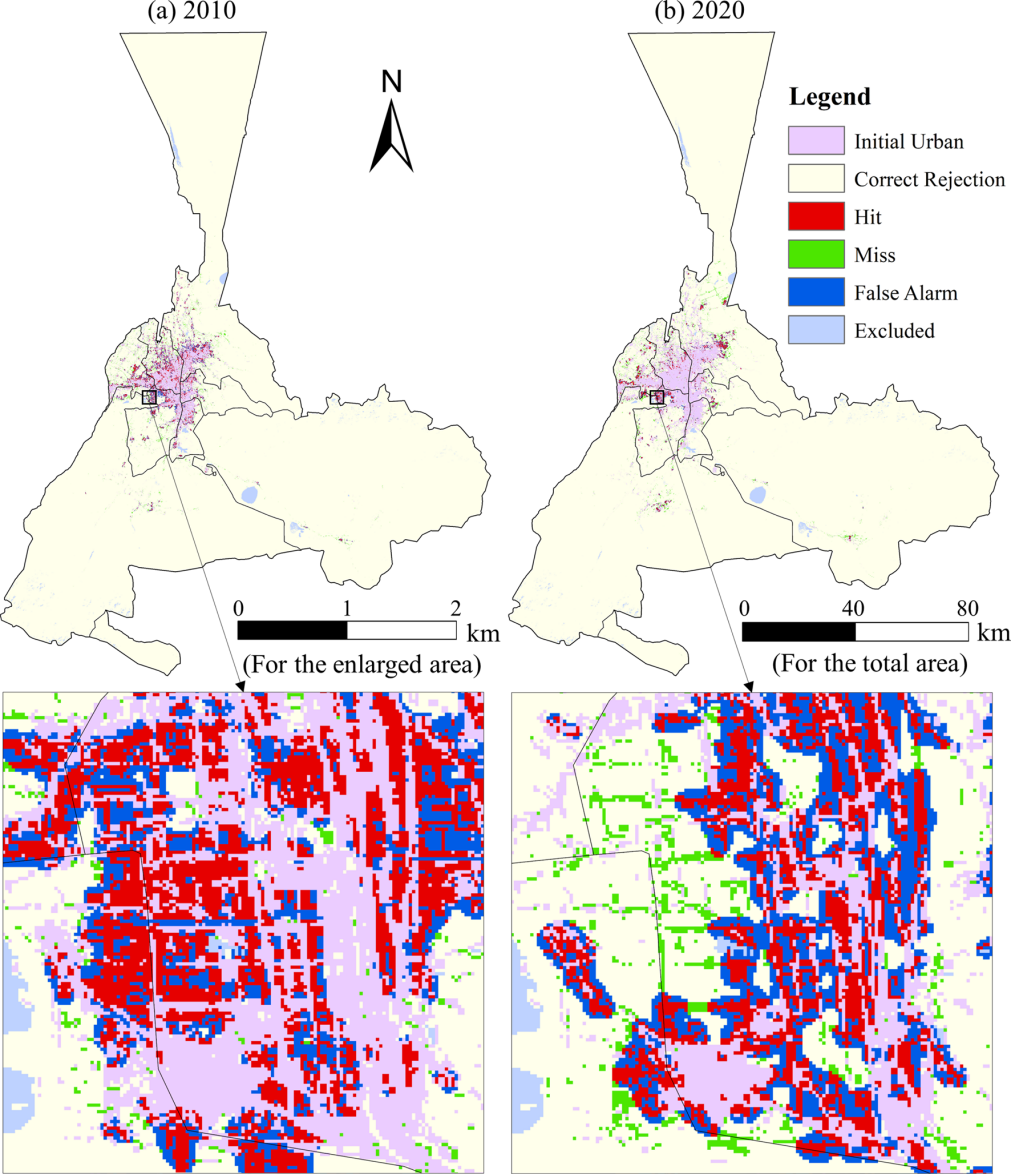
The FOM assessment of the simulated results produced by GOA-CA with fractions of the population (*kbest*) at 0.5. The map is created by ArcMap, version 10.8 (http://www.esri.com).

Figure 8 illustrates the assessment maps that show the distribution of hit cells, missing cells, and false cells with the highest modeling accuracy for the calibration and validation for 2010 and 2020. These maps were dominated by correct projections (hits), omissions (misses), and incorrect predictions (false alarms) for 2010 and 2020, with *kbest* set to 0.5. The zoomed-in portion shows that accurate simulations typically occur near the original urban area, while false alarms are more prevalent near accurate simulations. Missed areas of projection are typically observed in proximity to correctly simulated neighborhoods.

### Future scenario projections

**Fig. 9:**
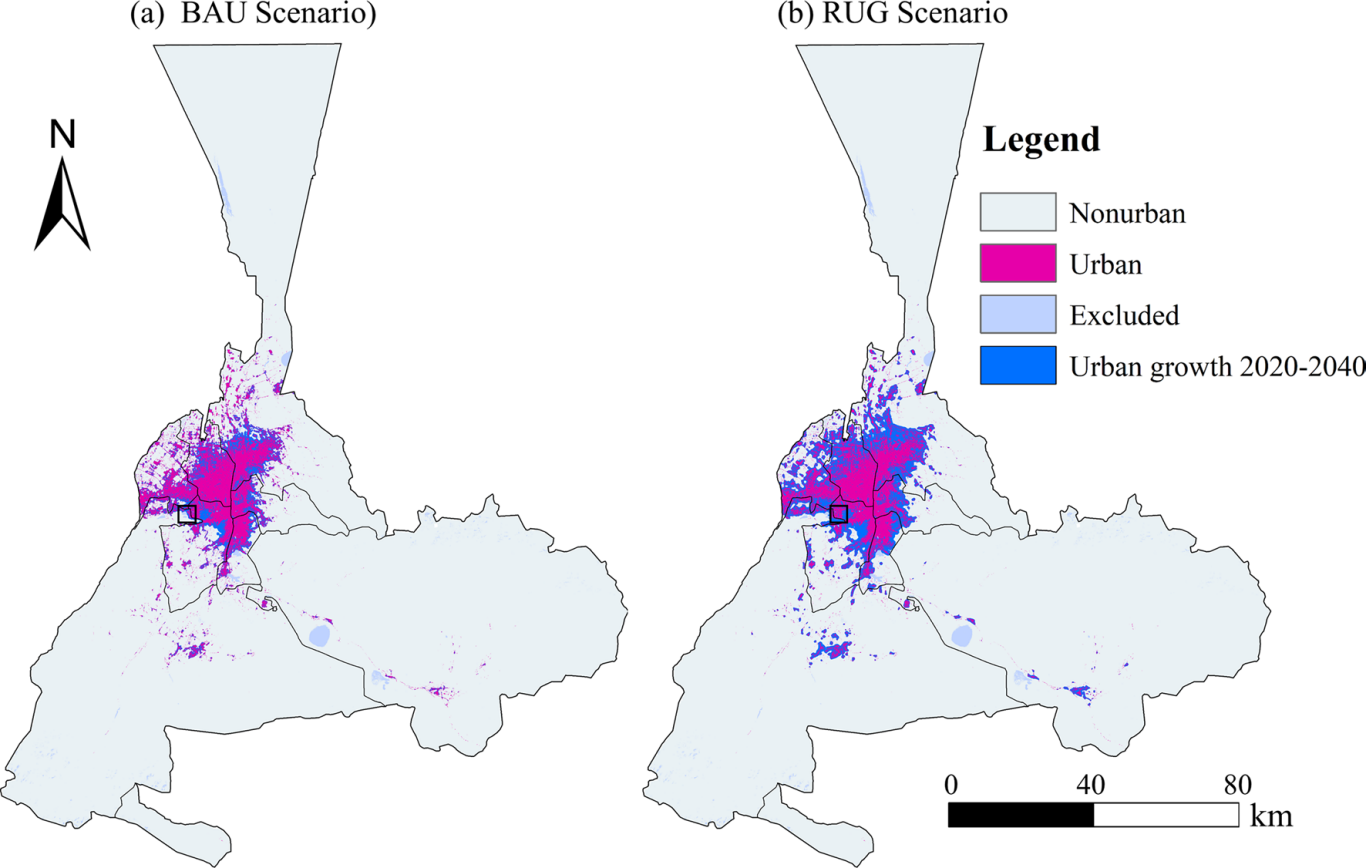
Urban growth in Urumqi for 2040 with two scenarios. The map is created by ArcMap, version 10.8 (http://www.esri.com).

We used the calibrated GSA-CA model based on the best-performing results from previous studies with fractions of the population (*kbest*) at 0.5 to project urban growth patterns in 2040 under two different scenarios (Fig. 9): (a) Scenario I (BAU Scenario): a business-as-usual (BAU) approach based on extrapolated urban growth rates from 2010 to 2020; and (b) Scenario II (RUG Scenario): a rapid urban growth (RUG) scenario, in which urban growth around the major urban centers is expected to accelerate.

In Scenario I, the same rate of urban growth in Urumqi is assumed as in the previous period, without allowing for potential changes in policy, infrastructure improvements, or economic conditions. In this scenario, urban growth is mainly dispersed around the original urban area, particularly in the Xinshi District and Midong District (Fig. 9a). In Scenario II, accelerated urban growth is foreseen, with the built-up area increasing by 1.5 times in the following 20 years. This scenario results in a triangular-shaped urban pattern concentrated in the peripheral areas of the urban area (Fig. 9b). Above all, the two urban growth patterns lead to two different urban scenarios, which provide valuable insights for urban planning and policy making in Urumqi.

## Discussion

The complex topography and special climate of arid regions exert a significant influence on urban growth and ultimately urban patterns^32,34^. Therefore, it is imperative to simulate accurately the dynamic urban growth in arid regions like Urumqi by considering the topographic features and climatic features^35^. Meanwhile, the effectiveness of modeling methods is crucial to reflecting the current urban conditions and projecting future urban scenarios^12^. Consequently, a meticulous selection of typical drivers for the study area and the optimization of modeling approaches are essential for generating different future land use and urban growth projections for arid regions, which in turn can help to formulate sound urban growth policies.

### The model performance and impact of heuristic parameterization on modeling

Regarding cellular automata-based urban growth simulation, many publications have applied different modeling approaches to simulate and project future scenarios for various kinds of areas. However, different modeling approaches perform differently in arid regions, and the same models perform differently in different arid regions^36^. The models including SLEUTH, CA-Markov, and FLUS are widely recognized for their ability to dynamically simulate the urban growth process by considering the influencing factors in a comprehensive way^35,37^. SLEUTH-Density model was developed to simulate the land density in the built-up area of Ajmer city, India, which has good performance^38^. Researchers used CA-Markov to investigate land use and land cover change in the desert region of Pakistan with an overall accuracy of more than 87%^39^. The overall accuracy of our modeling is over 98%, which is much higher than the simulations of many cities, confirming the superiority of our results^28^. This suggests that our method provides a commendable alternative for urban growth simulation and projection in arid regions.

Parameter settings for CA models targeting different study areas can vary significantly^12,28^. Fundamentally, GSA is a stochastic optimization algorithm that generates different simulation results when run multiple times with the same training data^40,41^. In our calibration of nine GSA-CA models with a parameter for the fraction of the population with the best fitness, we determined that the fraction of the population with the best fitness was 0.5. The study found that the modeling accuracy of the model improved slightly as the fraction of the population with the best fitness increased, and that beyond the 0.5 threshold, increasing the fraction of the population with the best fitness did not significantly improve GSA performance. It is therefore recommended that for regions like Urumqi, the fraction of the population with 0.5 may be the most effective parameter.

### The leading factors of Urumqi expansion and the suggestions based on the research

The CA parameters automatically identified by the GSA heuristic algorithm can account for the contribution of each factor to urban growth in arid regions. In this study, DEM is the most important factor inhibiting urban growth, which may be given Urumqi is surrounded by mountains on three sides, with more than half of the mountainous area, leading to a very limited amount of urban land^35^. City and town are the most important factors contributing to the promotion of urban growth, followed by road and railway, which suggests that for cities in arid regions, the location and transportation factors may have an impact on urban growth, and unplanned urban growth may exacerbate the further deterioration of drought.

The research findings have led to the formulation of several recommendations for urban management and planning in the city of Urumqi. Firstly, we would like to advocate an in-depth investigation into the dynamics of urban growth in arid regions to provide reliable data support for the sustainable development of cities in arid regions^1,42^. Secondly, it is proposed that local governments should formulate rational urban planning policies to improve land use efficiency^43^. Thirdly, given the limitations of terrain and climate conditions, urban infrastructure should be strengthened to enhance the radial links between cities^44,45^.

### Deficiencies and direction of future efforts

The study simulated and projected urban growth in an arid region based on the heuristic algorithm. The driving factors are time-dependent and would vary under different scenarios, we did not consider the effect of their changes on the model, and it may improve the modeling accuracy if their changes are incorporated into the model. In addition, the model requires a large amount of land use and driver factor as inputs, and the quality and reliability of these remotely sensed data will have an impact on model performance. The uncertainty in modeling results caused by the accuracy of data inputs can be reduced if higher-resolution data are available.

## Conclusions

Modeling and projecting urban growth in arid regions is essential for sustainable development. In recognition of the distinctive characteristics of cities in arid regions, we developed an innovative CA model (GSA-CA) using GSA to simulate and project urban growth in arid regions. We calibrated the GSA-CA model for Urumqi using datasets from 2000 to 2010, validated it using datasets from 2010 to 2020, and subsequently projected urban growth scenarios for 2040. The results showed that Urumqi's urban growth during the past 20 years occurred mainly in the Xinshi District, Tianshan District, Shaybak District, and the surrounding low-lying areas, showing a clear agglomeration pattern. The overall accuracy of the model in 2010 was 98.42%, with a FOM of 43.03%; in 2020, the accuracy was 98.52%, with a FOM of 37.64%, which fully proves the effectiveness of the novel model. Finally, we projected two potential scenarios for Urumqi in 2040, namely the BAU scenario and the RD scenario, to help improve urban planning and development strategies.

The model is capable of simulating urban growth and project future scenarios well in arid regions worldwide. Future research should focus on identifying and integrating key meteorological and ecological factors to improve the projection capability of CA models for urban growth in arid regions.

## Data Availability

The data we used is available at https://figshare.com/s/77dab4d8367d3b5a7673.
